# The Book World of Medicine and Science

**Published:** 1903-05-23

**Authors:** 


### The Book World of Medicine and Science.

Biographic Clinics. The Origin of the Ill-health of De Quincey, Carlyle, Darwin, Huxley, and Browning. By George M. Gould, M.D., Editor of *American Medicine*, Author of "An Illustrated Dictionary of Medicine, Biology, etc.," "Borderland Studies," etc. 223 pages, with frontispiece. (London: Rebman, Limited. 1903. Price 5s. net.)

Dr. Gould is well known on both sides of the Atlantic as a journalist of unlimited resource and a writer of considerable literary distinction, and in his latest volume he certainly well maintains his high reputation for originality, ingenuity, and artistic facility. Dr. Gould is of opinion that medical science has hitherto been too restricted in its analysis of disease. The average medical man busies himself with the patient's complaint upon the day of consultation, or during the time of the special illness, and casts but few glances "before and after." When the physician has succeeded, or perhaps has failed, in his special and immediate duty, he usually thinks how he may treat other patients suffering from the same complaint, and he writes of his experience, and of his success or failure, in such cases always concerning the single disease in question and its cure. The philosophic physician, Dr. Gould seeks to show, generally studies the pathogeny of a single type apart from all individual origin and relations: the suffering, even the existence, of the particular patient is not in the attention. Our author, no doubt quite rightly, contends that the wise physician will consider the patient's life problem, studying the subject as a whole, all of his diseases, past, present, and to come, and carefully ascertaining what relation they bear to the daily life and sociological conditions. Each patient rightly viewed is a history and a prophecy; and Dr. Gould illustrates his method by reference to records of the life, labours, and lamentations of five master minds. Clinical details of the ailments which handicapped and grievously fettered the working capacity and limited the powers of happiness of such men as De Quincey, Carlyle, Darwin, Huxley, and Browning are here presented in systematically arranged extracts from the letters and biographical records of these distinguished men. Doubtless the aches and smarts, the growls and grumbles, of life's little ailments might by most of us be extended into an amusing catalogue if folly would induce us to prepare our autobiography, or an enemy could undertake the elaboration of our life history. Interesting and highly suggestive as Dr. Gould's method may be for the collection of clinical data, we cannot but help feeling that it is not altogether free from serious dangers. In the study of the disabilities of his selected characters Dr. Gould arrives at the conclusion that each owed his trouble chiefly, if not solely, to ocular deficiencies. Thomas De Quincey "had myopic astigmatism of some anomalous and anisometropic variety"; Thomas Carlyle's lamentations originated in "astigmatism . . . which no common optician's lenses could help"; the symptoms of Charles Darwin, "vomiting and other functional digestional disorders, insomnia, vertigo, headache, apathy, and neurasthenia, are precisely those which the best American oculists find are the most common symptoms of this [compound hyeropic astigmatism] refractive anomaly of the eyes"; Thomas Huxley, too, "had far-sighted astigmatism"; and even Robert Browning's sorrows arose from "an astigmatism which could no longer be neutralised by the muscle of accommodation." Dr. Gould concludes his "Biographic Clinics" with several interesting studies on biliousness and headache, the physiology of vision, astigmatism and eye-strain, and the responsibilities of medical men in relation to the investigation and management of such cases as have been under consideration. Dr. Gould's book is peculiarly attractive and rich in suggestion. It presents on every page evidence of painstaking research and much thought. It will undoubtedly do good in drawing attention to only too readily neglected conditions; but, in spite of all, we cannot but feel that the case has been weakened by an over-insistence on the ophthalmic specialist's point of view.

Ocular Therapeutics, According to the Most Recent Discoveries. By Dr A. Darier. Translated by Sydney Stephenson, M.B., C.M. (London: Churchill. Large 8vo. pp. 278. Price 10s. 6d.)

Those members of the medical profession in this country who do not keep themselves abreast of French literature will have much reason to be grateful to Mr. Stephenson for his very clear and pleasing translation of the second edition of Dr. Darier's "Leçons de Thérapeutique Oculaire." Dr. Darier has long been known as a painstaking searcher after new therapeutical agents, and as a trustworthy investigator of their merits and defects; and the lectures translated by Mr. Stephenson give a lucid picture of the generally satisfactory results which his author has attained. Dr. Darier is perhaps best known in this country as the introducer of acoine, the agent by which the sub-conjunctival injection of mercuric solutions has been rendered practically painless, and of dionine, by which the speedy resolution of many forms of intense and painful ocular inflammation has been brought about. The work before us contains full accounts of both these agents, of their uses, and of the methods of applying them, as well as information of the same kind about others which as yet have made but little way in this country. It is not too much to say that Dr. Darier and his fellow workers have revolutionised the treatment of eye disease, have introduced many new and valuable medicines into ophthalmic practice, and have greatly improved the methods of employing many old ones. No practitioner who wishes to do the best that is possible for his patients should neglect to acquaint himself with these improvements, and we are able to say from personal experience that the value of many of them is by no means overstated. The translator has added a few notes, mostly very brief, but always useful and to the purpose.

Practical Hints for Travellers in the Near East. By E. Reynolds Ball, F.R.G.S. (London: Marlborough and Co. 1903. 8vo. pp. 140. Price 2s.)

Mr. Reynolds Ball has obligingly compiled this little handbook for Eastern travellers in the hope that it may serve as a companion volume to the guide books. In the brief compass of 140 pages, Morocco, Algeria, Tunis, Egypt, Syria, Palestine, Turkey, Greece, Cyprus, and Malta are dealt with specifically, while general hints and suggestions are given that may be found "serviceable over a much more extended field of travel." A section is devoted to medical hints, and we are informed by the author, Mr. Ball, that this section has been read and approved by a London medical man of high standing. An important feature of this little work is the care with which exactly the proper articles for "travellers in the near East" to purchase before starting on their adventures are indicated; and it doubtless will not escape the notice of such travellers that, in the advertisement pages are given full particulars of each one of the articles recommended in the text by the author.

